# Supplementary material for: Improvement of Water and Nitrogen Use Efficiencies by Alternative Cropping Systems Based on a Model Approach
Source: Plants (Basel). 2023 Jan 29;12(3):597. doi: 10.3390/plants12030597 (PMC9919017; doi:10.3390/plants12030597)
Supplement: Supplementary file 1 [file plants-12-00597-s001.zip › plants-2166866-supplementary.pdf]

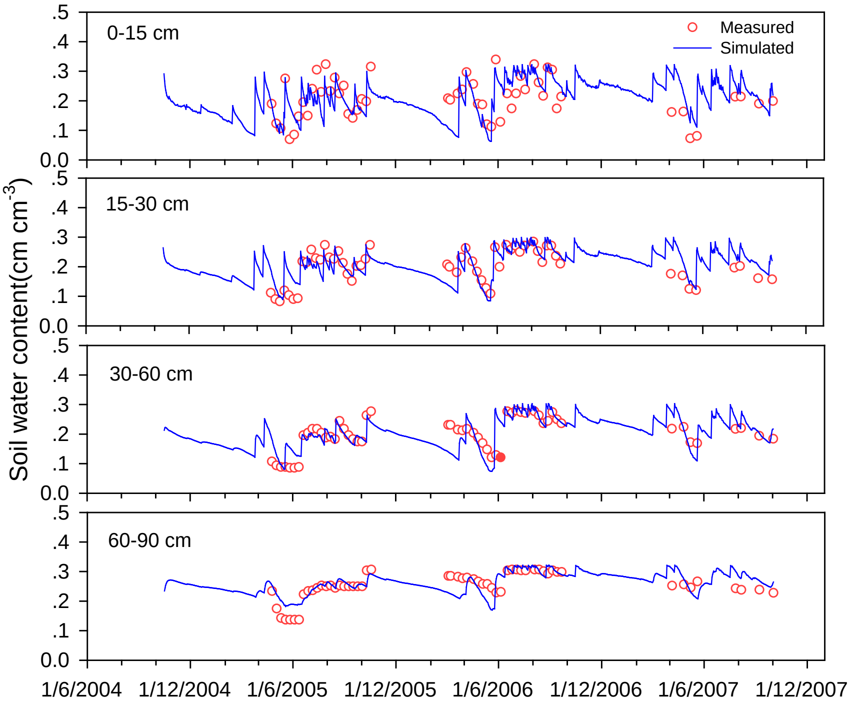

Figure S1. Comparison of simulated and measured soil water content at different depths for 2H1Y_FP.

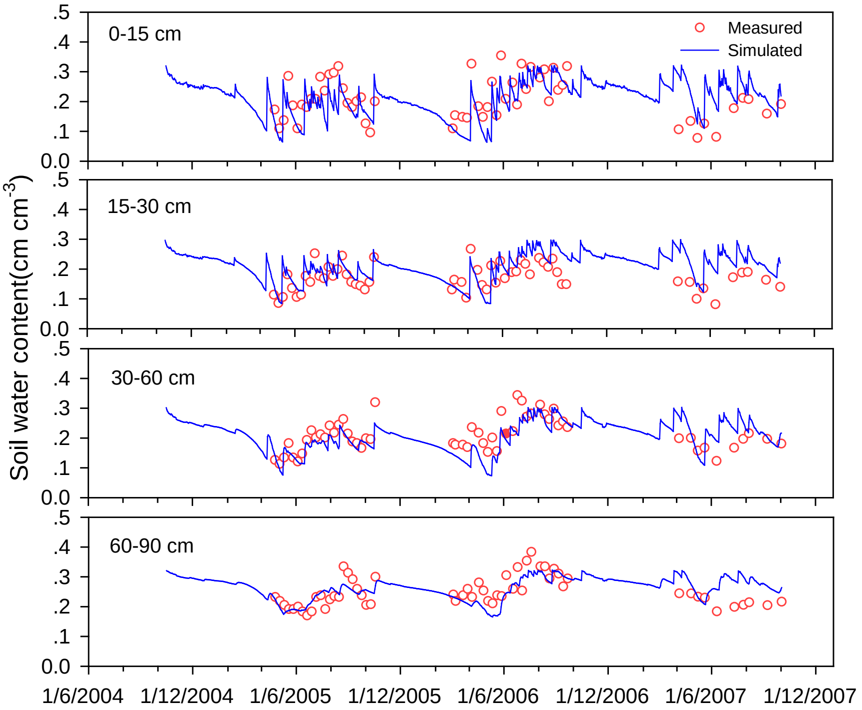

Figure S2. Comparison of simulated and measured soil water content at different depths for 2H1Y_WQ.

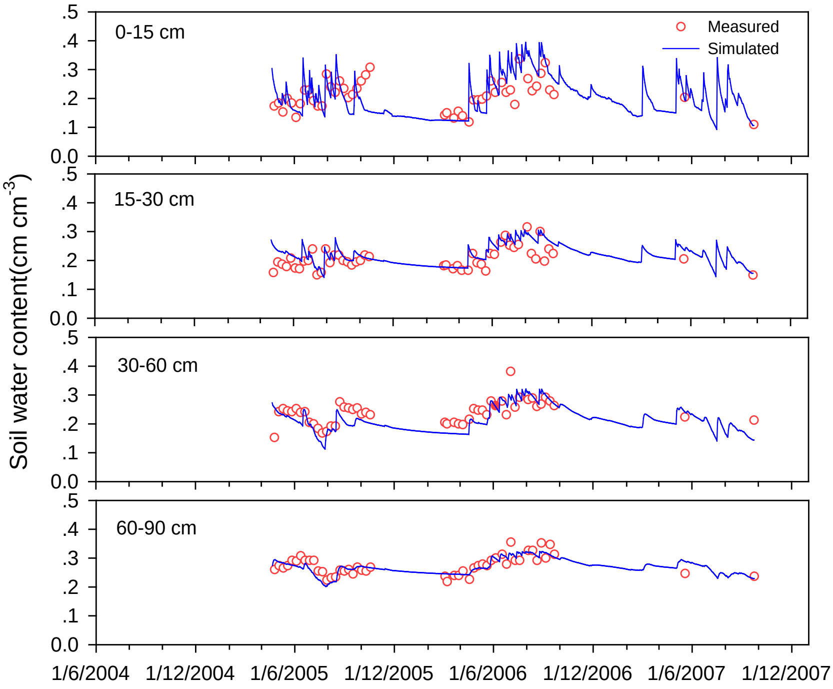

Figure S3. Comparison of simulated and measured soil water content at different depths for 1H1Y.

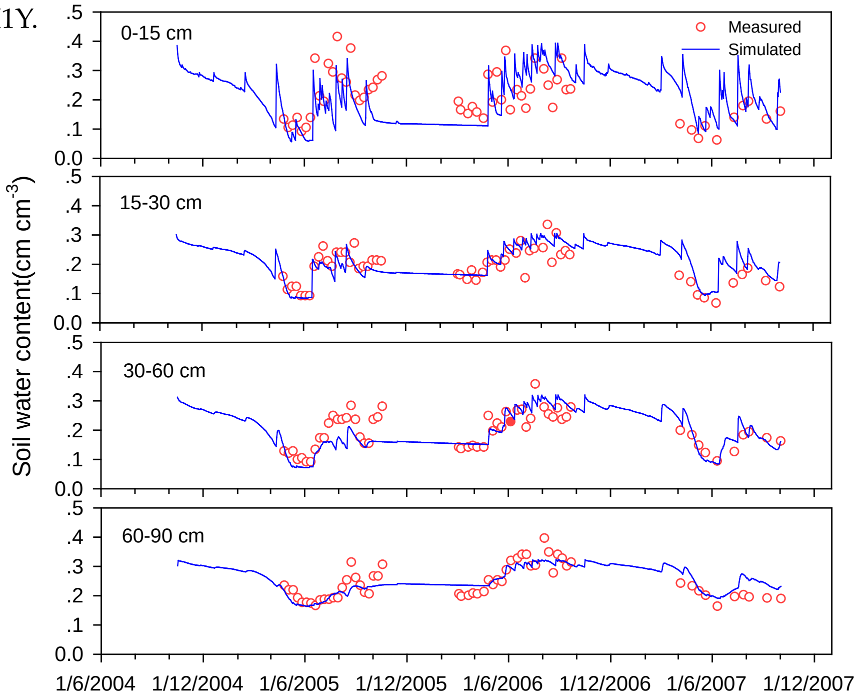

Figure S4. Comparison of simulated and measured soil water content at different depths for 3H2Y.

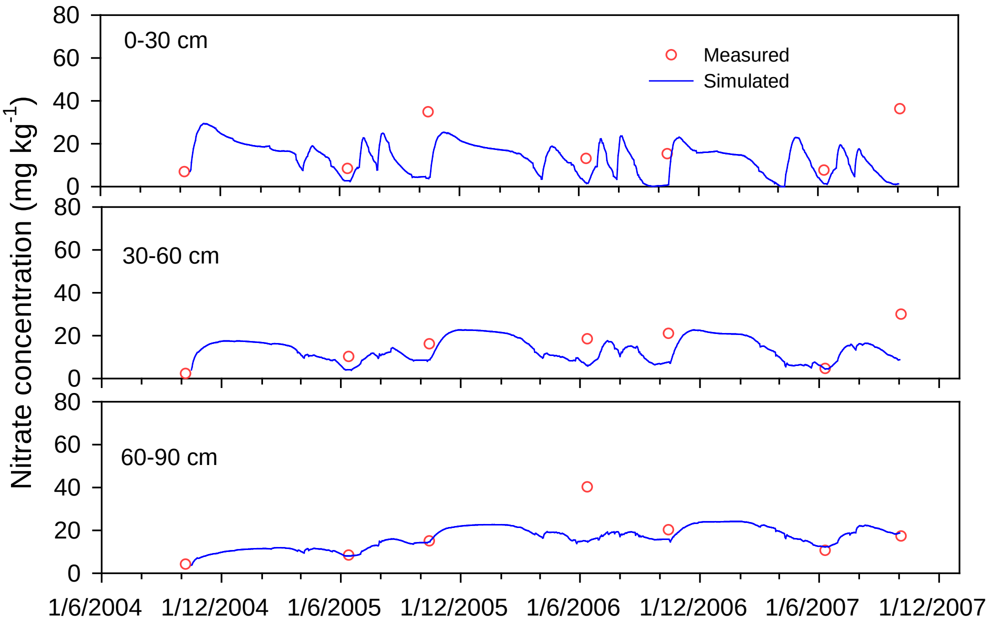

Figure S5. Comparison of simulated and measured soil nitrate N concentration at different depths for 2H1Y_FP.

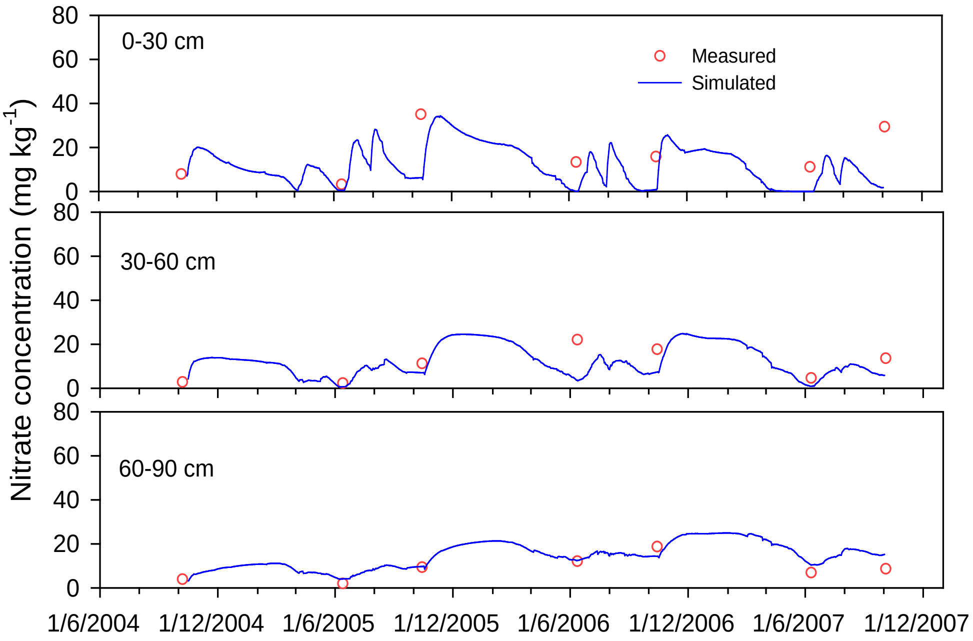

Figure S6. Comparison of simulated and measured soil nitrate N concentration at different depths for 2H1Y_WQ.

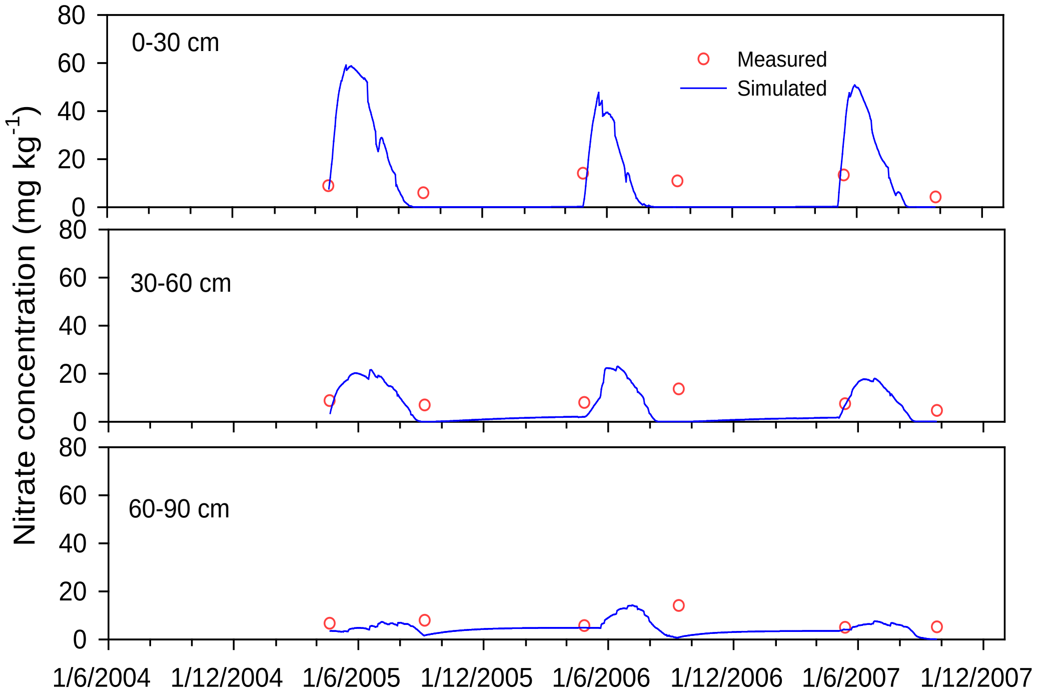

Figure S7. Comparison of simulated and measured soil nitrate N concentration at different depths for 1H1Y.

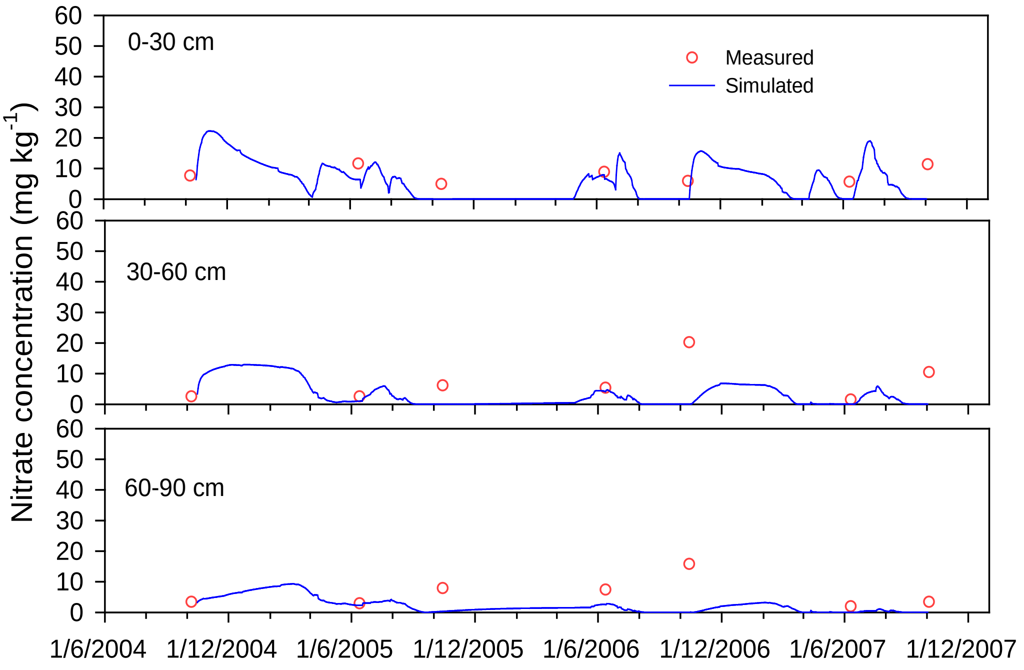

Figure S8. Comparison of simulated and measured soil nitrate N concentration at different depths for 3H2Y.
